# Chitosan Fibers Modified with HAp/β–TCP Nanoparticles

**DOI:** 10.3390/ijms12117286

**Published:** 2011-10-25

**Authors:** Dariusz Wawro, Luciano Pighinelli

**Affiliations:** Institute of Biopolymers and Chemical Fibers—IBWCh, Sklodowskiej-Curie 19/27, 90-570 Lodz, Poland; E-Mail: pighinelli@hotmail.com

**Keywords:** chitosan fibers, calcium phosphate, nanoparticles, mechanical properties

## Abstract

This paper describes a method for preparing chitosan fibers modified with hydroxyapatite (HAp), tricalcium phosphate (β-TCP), and HAp/β-TCP nanoparticles. Fiber-grade chitosan derived from the northern shrimp (Pandalus borealis) and nanoparticles of tricalcium phosphate (β-TCP) and hydroxyapatite (HAp) suspended in a diluted chitosan solution were used in the investigation. Diluted chitosan solution containing nanoparticles of Hap/β-TCP was introduced to a 5.16 wt% solution of chitosan in 3.0 wt% acetic acid. The properties of the spinning solutions were examined. Chitosan fibers modified with nanoparticles of HAp/β-TCP were characterized by a level of tenacity and calcium content one hundred times higher than that of regular chitosan fibers.

## 1. Introduction

Fibers made of chitosan are widely used in new generation biomaterials. The formation of chitosan fibers is a rather simple process usually accomplished by wet spinning from a polymer solution. A variety of fibrous chitosan materials are available, ranging from fine microfibrids [1] to very strong fibers and pseudo-dry-spun fibers [2] and multifilament chitosan yarns [3,4]. Wet spinning from a solution of the polymer enables modifications by adding various functional substances to the solution, notably nanoparticles, carbon nanotubes (CNT) [5–7], nanosilver [8], calcium phosphates [9], calcium sulphite [10] and a number of polymers such as fibroin and keratin proteins [11,12].

Chitosan lends itself to use in biomaterials thanks to properties such as its biodegradability, biosorption and ability to accelerate wound healing.

To confer functionality upon chitosan fibers, they are frequently coated with other biomaterials such as collagen or calcium phosphates [13–17]. Following coating, they are used to reinforce composite implants containing hydroxyapatites (HAp) [18]. Calcium phosphates are known for their compatibility, osteoconductivity and easy absorption by the human body, which makes them suitable for the preparation of composite dental implants [19] and, in combination with chitosan, artificial bones [20,21].

Chitosan multifilament yarn, which is used in the construction of textile scaffolds or as culture medium for cell growth, requires the addition of calcium phosphates like HAp or β-TCP and has been successfully used in medical applications such as artificial bones, cartilage and glues for hard tissue engineering [3,13–15,20–23].

Calcium phosphates are not easy to dissolve in chitosan solutions; dissolution depends on their chemical composition, crystallinity and dissolving conditions. For this reason HAp nanoparticles can be used in the preparation of chitosan gels [24] or can be introduced into the fiber matrix.

Calcium phosphates HAp and β-TCP have different dissolving properties, and hence their release from composites or chitosan fibers may differ. For this reason they are usually employed in a mixture to maintain a favorable calcium-to-phosphorus ratio of 1.67 [25].

The aim of this study was to prepare novel chitosan fibers containing nanoparticles of calcium phosphates for medical use, particularly for textile scaffolds and cell growth. We also investigated how nanoparticles of β-TCP, HAp and of the complex HAp/β-TCP affect the rheology of the chitosan spinning solution, spinning conditions and mechanical properties of the resulting HAp/β-TCP-modified fibers.

## 2. Results and Discussion

### 2.1. Preparation of Chitosan Solutions Containing β-TCP, HAp and HAp/β-TCP

A 5.16 wt% aqueous chitosan solution (A) was prepared in 3.0 wt% acetic acid. The solution was blended with a chitosan solution containing β-TCP, HAp, or a mixture of solutions containing HAp/β-TCP nanoparticles in the appropriate proportions. Table 1 presents the properties and the composition and some properties of these solutions.

The 5.16 wt% chitosan solution (A) had a temperature of 52 °C and a high dynamic viscosity of 19000 Pa. The dissolution of chitosan and the blending of the particular solutions caused no problems. Admixing of the solution with calcium phosphate resulted in a decrease in the concentration of the acid and chitosan and in the dynamic viscosity. Small particles of calcium phosphate (β-TCP) were found in the chitosan solutions upon microscopic observation. A clear, stabile chitosan solution suitable for spinning was obtained by blending solutions A and B (code MCT 6).

Comparison of microscopic images of the chitosan solutions denoted Chit 58 (A) and MCT 6 indicated that the introduction of the chitosan solution containing β-TCP nanoparticles reduced the number of undissolved particles in the MCT 6 solution.

Hydroxyapatite is less soluble than β-TCP; this was apparent in the course of preparing solution C, when only a small amount of HAp dissolved while the majority appeared as an opalescent suspension.

Blending of the HAp-containing chitosan solution with the chitosan spinning solution (A) only slightly improved the solubility of HAp in the chitosan acetate solution. Hence, the chitosan acetate solution denoted MCT 7 contained HAp particles with a diameter of up to a few micrometres. Though not negating the solution’s spinnability, this feature may negatively influence the quality of the prepared calcium-containing fiber.

The reverse was the case when chitosan acetate solution was mixed with chitosan B and C solutions containing HAp and β-TCP (solution B contained 0.9 wt% hydrochloric acid). The result was the complete dissolution of the hydroxyapatite and a clear solution denoted MCT 11.

It is likely that mixing of the chitosan solution containing HAp with the chitosan solution containing β-TCP in hydrochloric acid at a ratio of 2:1 provided adequate conditions for the dissolution of HAp. The dissolution of HAp in chitosan solution is the subject of further investigations and of a patent application [26]. The mechanism of salts and amino acids upon the dissolution of HAp has been reported previously [27]. Mixing of solutions B and C in the ratio 1:1 did not cause HAp to dissolve (MCT 8).

Microscopic analysis of the MCT 7 and MCT 11 chitosan acetate solutions confirmed the presence of HAp particles in the MCT 7 solution, while the B/C mixture (MCT 11) clearly contained HAp/β-TCP nanoparticles. The microscopic image of the MCT 11 solution was optically pure. The chitosan solution (B) containing β-TCP nanoparticles was also clear and optically pure. Hydroxyapatite added to chitosan acetate solution (C) produced a suspension with only some of it going into solution. Agglomerates with a size of 1500 nm that are present in the spinning solution may negatively influence the mechanical properties of the spun fibers. The situation was slightly improved when mixing the chitosan B/C solution with the chitosan A solution in a 1:1 ratio, which resulted in a chitosan solution (MCT 8) with fewer HAp particles. Another solution of the B/C mixture was prepared with more of the β-TCP particle-containing chitosan solution (2:1). During the blending of the HAp and β-TCP particle-containing chitosan solutions, the HAp particles dissolved immediately, resulting in a clear solution (MCT 11). Comparison of the images of the chitosan solutions MCT 7 and MCT 11 revealed that mixing the HAp- and β-TCP-containing chitosan solutions had a beneficial effect by limiting the size of the nanoparticles within the range of 12.8–58.0 nm in MCT 11.

**Figure 1:**
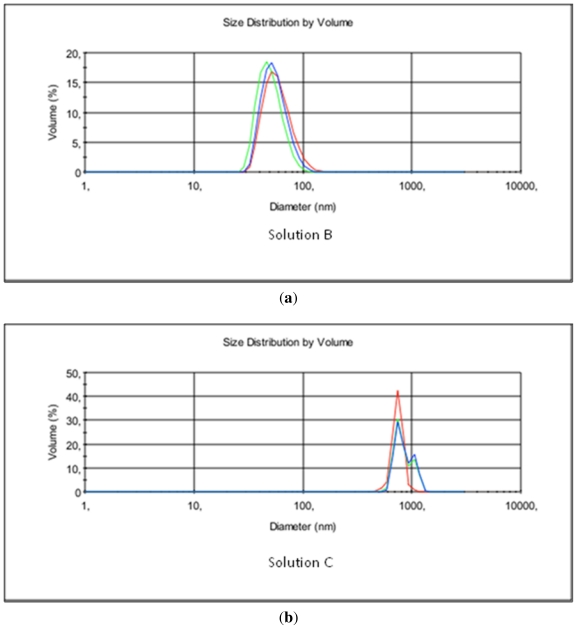

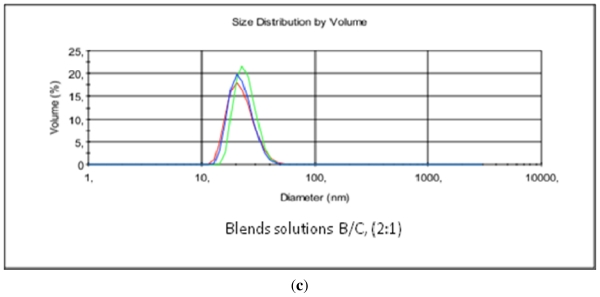
(**a**) Particle size distribution of β-TCP in chitosan solution in hydrochloric acid (solution B); (**b**) HAp particles in chitosan acetate solution (solution C); (**c**) The HAp/β-TCP blend in chitosan (solution B/C).

Figure 1(a–c), presents the particle size distribution in the chitosan solutions containing HAp or β-TCP and HAp and β-TCP.

Table 2 shows selected parameters of the solutions analyzed by means of the ZetaSizer analyzer. The results presented in Table 2 and Figure 1 confirm. The smallest nanoparticles found in the B/C mixture were much smaller than those in chitosan solutions B and C before blending.

The chitosan solutions characterized in Table 2 were prepared for the spinning of chitosan fibers. Addition of the calcium phosphate nanoparticles to the chitosan solutions resulted in a slight drop in the chitosan content and concentration of acetic acid, an insignificant increase in the hydrochloric acid content in the blended mixture, and a radical decrease in the apparent dynamic viscosity. The Zeta potential showed greater stability for the solution MCT-11 (solution A + B/C(2:1)) in this ratio than for other solutions.

### 2.2. Rheology of Chitosan Solutions Modified with HAp/β-TCP Nanoparticles

**Figure 2:**
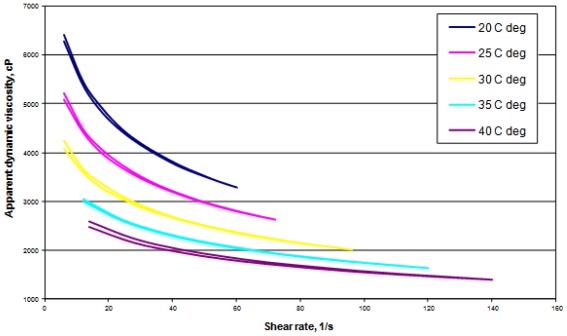
Dependence of the apparent dynamic viscosity on the shearing rate and temperature of the acetate chitosan solution modified with HAp/β-TCP (MCT 11).

The rheology of chitosan solution MCT 11 modified with HAp/β-TCP nanoparticles was examined at 20, 25, 30, 35 and 40 °C. The impact of shearing speed and temperature upon the dynamic viscosity of the chitosan solution modified with calcium phosphate is presented in Figure 2. The flow curves were drawn for the rise and fall in shearing speed.

It can be inferred from the curves that chitosan modified with HAp/β-TCP nanoparticles has the attributes of a non-newtonian fluid. In the examined temperature range, the viscosity of the solution decreased with increasing shearing speed. The flow curves overlapped with each other both at increasing and decreasing shearing speed. Chitosan solutions thus showed the features of a pseudo-plastic fluid (they are diluted by shearing) typical of polymer solutions. An increase in temperature from 20 to 25 °C distinctly reduced the dynamic viscosity. Further increases in temperature and shearing speed caused much greater decreases in viscosity.

Given that fibers are spun from solutions of chitosan acetate and that the spinning process takes a relatively long time, the stability of solutions as confirmed by the rheological examinations is of particular importance.

### 2.3. Investigation into the Spinning of Chitosan Fibers Modified with HAp, β-TCP and HAp/β-TCP

To allow comparison, regular chitosan fibers and those modified with calcium phosphate were spun under the same conditions at a spinning speed of 31.0 m/min and a draw ratio of 34%. The fibers were spun into a coagulation bath containing aqueous 3.0% NaOH without ethanol. The spinning process of both regular chitosan fibers and those modified with β-TCP, HAp and HAp/β-TCP was stable in the adopted conditions with no disturbance in the spinneret zone. The addition of calcium phosphates to the spinning solution had a beneficial effect on spinning stability, with fewer breaks in the elementary filaments.

### 2.4. Mechanical Properties of Chitosan Fibers Modified with HAp, β-TCP and HAp/β-TCP

The mechanical properties of chitosan fibers modified with HAp/β-TCP are presented in Table 3. The impact of the calcium phosphate on the mechanical properties of the fibers varied depending on the type and amount of additive used. The addition of β-TCP had little effect on tenacity (MCT 6) while the same amount of HAp reduced both the tenacity and elongation (MCT 7). The large amount of HAp agglomerates in the fibers may be the underlying cause. Blending of the chitosan solutions with HAp and β-TCP led to an improvement in the solution with higher amounts of calcium phosphates (some of the HAp particles dissolved), with a slight increase in fiber tenacity (MCT 8). Doubling of the amount of chitosan solution B (containing β-TCP) in the mixture led to a homogenous solution of HAp/β-TCP (MCT 11) nanoparticles along with the highest level of calcium phosphates, and resulted in fibers with the same tenacity as that of regular chitosan fibers.

When dried in a loose bundle, chitosan fibers modified with HAp/β-TCP (MCT 11) did not stick to each other; a feature that meant a finishing agent was not required.

An advantage of chitosan fibers containing HAp/β-TCP (MCT 11) nanoparticles is their high tenacity and low coefficient of variability of breaking force in wet conditions in comparison with other fibers.

### 2.5. FTIR Examination of Chitosan Fibers Modified with HAp, β-TCP and HAp/β-TCP Nanoparticles

**Figure 3:**
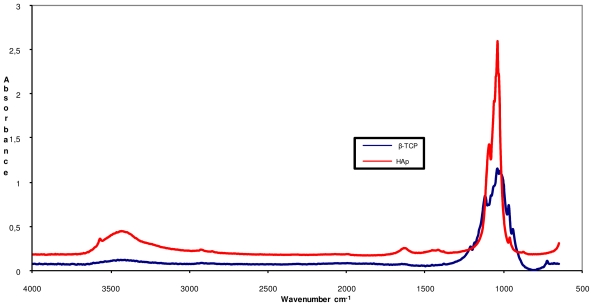
FTIR spectrum of the commercial HAp and β-TCP.

The main peaks in energy vibration identified in the β-TCP and HAp powders are shown in Figure 3. The functional groups of orthophosphate (PO_4_ ^3−^), hydroxyl (–OH) and phosphate (HPO_4_ ^2−^), the latter one in trace amounts, are characteristics of apatite materials. Trace amounts of (CO_3_ ^2−^) groups were observed at 1428 cm^−1^, for both β-TCP and HAp, indicating that in some commercial calcium phosphate powders, CaO and Ca(OH)_2_ are added to achieve the ideal stoichiometric proportion between Ca/P [23,25,28–30]. In the Figure 3 show the FTIR spectrum of the commercial calcium phosphate.

The absence of bands at 460 and 740 cm^−1^ and an isolated band at 600 cm^−1^, characteristic of β-TCP, indicate that the starting material was composed of β-TCP only. This calcium phosphate is easily identified by a broad band at 900–1200 cm^−1^, and the presence of a peak at 724 cm^−1^, characteristic of the symmetric mode of (P–O–P), the distortion of P–O. The peak at 1211 cm^−1^ is characteristic of the non-degenerate deformation of hydrogen groups (H–OPO_3_, O–PO_3_, HPO_4_ ^2−^), which may reflect the interaction with water molecules in the structure [23,29,31,32–34].

Figure 3 shows the spectrum of HAp with a peak at 839 cm^−1^ that corresponds to the deformation modes of the phosphate group (O–P–H) bonds, which are associated with the energy levels and the rotational type of (O–H) bond. The (–OH) group peaks are also apparent at 3570 cm^−1^ and 3464 cm^−1^, reflecting the way they stretch.

The low intensity peak visible at 962 cm^−1^ corresponds to the non-degenerate symmetric stretching of P–O bonds in the phosphate groups. The peak at 1040 and 1093 cm^−1^ represent the asymmetric stretch modes of the P–O bonds in the phosphate groups [23,31–34].

**Figure 4:**
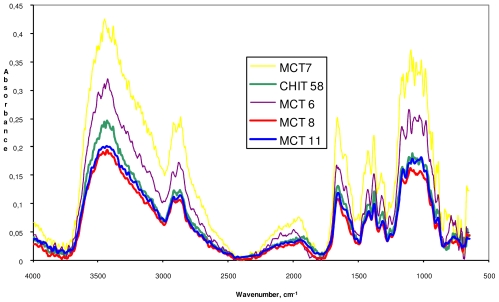
FTIR spectra of chitosan fibers (Chit 58) and modified β-TCP (MCT 6), and those modified with HAp (MCT 7), HAp/β-TCP (1:1) (MCT 8) and HAp/β-TCP (1:2) (MCT 11).

Figure 4 shows the FTIR spectra of the MCT 7 HAp composite. spectrum exhibits characteristic bands such as: a strong and broad peak between 1032 cm^−1^ and 1086 cm^−1^ and 1160 cm^−1^ that reflects the skeletal vibrations stretch of the saccharide structure and a peak at 1559, 1543 cm^−1^ attributed to the free primary amino group (NH_2_), amide I (1637 cm^−1^) indicating that chitosan used in this research is partially deacetylated (83.2%), amide II (1559 cm^−1^) and amide III (1319 cm^−1^). The anti-symmetric stretch bridge C–O–C (1160 cm^−1^), stretch N–H (3298, 3500 cm^−1^), stretch O–H (3445 cm^−1^) and the main peaks of energy vibration (1032, 1086 cm^−1^) identified the chitosan. The peak at 1243 cm^−1^ represents the free amino groups and the C2 position of glycosamine [23,25,27,30–32,35]. The main peaks of energy vibration identified in HAp are characteristic of the functional groups of orthophosphate (PO_4_ ^3−^) at 1100 cm^−1^, hydroxyl (–OH) at 3700 and 2600 cm^−1^ and phosphate (HPO_4_ ^2−^) at 1000 cm^−1^.

The band in the region from 1590 to 1635 cm^−1^ and the peaks around 3400 cm^−1^, corresponding to the (–OH) stretching absorption band, suggests the presence of water molecules in the sample [19,25,27,31,36]. The chemical interactions between the inorganic and organic constituents in the composite probably take place via ionic bonding between (Ca^2+^) and phosphate groups and the amino group of chitosan, confirming the positive charge of chitosan and the negative charge of β-TCP [21,23,25,30,31,35].

All band features were similar to those described by Brugnerotto *et al*. [21,37] and were present in all samples investigated, showing that all have basically the same functional groups.

The exact calcium phosphate phase formed under acidic conditions is influenced by the specific anions present. In some cases, unusual phases may be formed under acidic conditions, such as CaCl_2_·Ca(H_2_PO_4_)_2_·2H_2_O at low pH or in the presence of HCl. The pH of the chitosan acetate solution was typically around 4 to 5. In this regime, HAp is expected to show good solubility and may exhibit phase instability [30,35].

High turbidity was observed in solutions containing HAp, which indicates a strong adsorption interaction between chitosan and the HAp surface. Chitosan has a relatively high zeta potential and can enhance the stability of HAp [30,35].

### 2.6. Morphology and Chemistry of Chitosan Fibers Modified with HAp, β-TCP and HAp/β-TCP Nanoparticles

The WRV of wet-spun regular- and HAp- and β-TCP modified chitosan fibers ranged from 150% to 331% depending on the calcium phosphate content.

MCT 8 had the highest WRV of 331%, which may be attributed to the high content of apatite agglomerates capable of forming water-retaining pores. The amount of calcium phosphate in the chitosan spinning solutions was estimated according to the calcium and ash content (Table 4).

**Figure 5:**
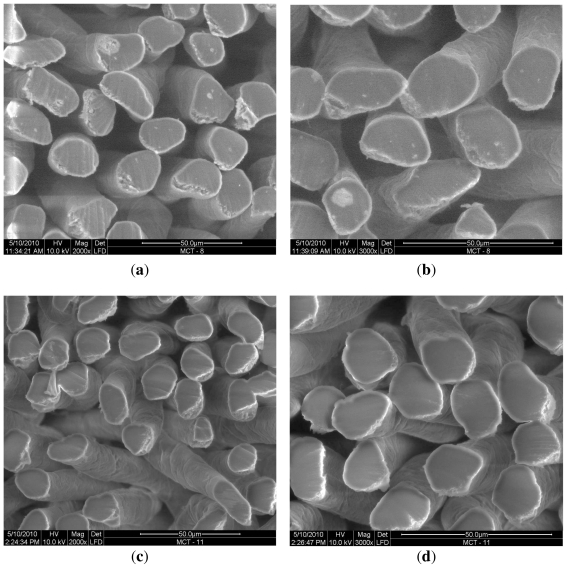
(**a**,**b**) SEM images of the surface and cross-section of chitosan fibers modified with HAp/β-TCP (MCT 8); (**c**,**d**) chitosan fibers modified with HAp/β-TCP nanoparticles (MCT 11).

The amount and size of HAp/β-TCP particles has a significant influence on the shape of the fiber cross-section. In MCT 11 it is oval and uniform with an undeveloped brim. The surface is characteristic of chitosan fibers with distinct recesses and grooves. The white spots that can be seen in the fiber cross-sections in Figure 5(a,b) represent HAp/β-TCP agglomerates. In contrast, chitosan fibers spun from solutions containing HAp/β-TCP nanoparticles have a pure cross-section with no traces of anything other than the fiber-forming polymer substances (Figures 5(c,d)).

## 3. Experimental Section

The following materials were used:

Tri-calcium phosphate (β-TCP) (Ca_3_(PO_4_)_2_), Hydroxyapatite (Ca_10_(PO_4_)_6_OH)_2_, supplied by Sigma Aldrich, Germany.

Glycerol as a plasticizer (C_3_H_8_O_3_), 99% pure, Sigma Aldrich, Germany.

Hydrochloric acid 37.8% pure, manufactured by Fluka, Germany.

Acetic acid 80% and NaOH made by POCh S.A., Gliwice, Poland.

Chitosan derived from the northern shrimp (Pandalus borealis), supplied by Primex Co., Norway. Table 1 shows some of the characteristics properties of chitosan such as Viscometric average molecular mass [kD] 342; Moisture content [%] 5.58; Water retention value [%] 43.5; Dynamic viscosity, 1 % chitosan in 1% acetic acid at 20 °C[CP] 63.1; Degree of deacetylation [%] 83.2; Ash content [%] 0,40; Nitrogen content [%] 6.84.

### 3.1. Preparation of Chitosan Spinning Solution Containing HAp, β-TCP and HAp/β-TCP Nanoparticles

A 5.0 wt% weight chitosan solution (A) was prepared in aqueous 3.0 wt% weight acetic acid. The dissolution of chitosan took 60 minutes, during which time glycerol was introduced to the solution to a volume of 10%. After filtration and deaeration at ambient temperature, the chitosan solution was blended with a 2.0 wt% chitosan solution containing β-TCP particles (solution B), a chitosan solution containing HAp (solution C) or a mixture of chitosan solutions containing HAp/β-TCP nanoparticles (solution B/C). Solution B was an aqueous 2.0 wt% solution of chitosan in 0.9 wt% solution of hydrochloric acid with a 2.0 wt% weight content of β-TCP nanoparticles. Solution C was a diluted aqueous 2.5 wt% chitosan solution in 1.5 wt% acetic acid containing HAp particles at a concentration of 2.0 wt%.

### 3.2. Wet Spinning of Chitosan Fibers Containing HAp, β-TCP and HAp/β-TCP Nanoparticles

Chitosan fibers modified with HAp, β-TCP and HAp/β-TCP nanoparticles were prepared by wet spinning on a pilot line equipped with a spinning head holding a rhodium/platinum spinneret with 150 holes, each having a diameter of 0.08 mm. The spinning solution is 35 °C, the same as a coagulation bath (aqueous 3.0 wt% sodium hydroxide at 35 °C). The spun fibers were washed first in a water bath at 40 °C then in a water-ethanol (60% v/v) bath and were then dried without tension in a loose bundle. The spinning speed for the chitosan fibers modified with HAp/β-TCP nanoparticles was 31 m/min.

### 3.3. Analytical Methods

The rheological properties of the chitosan solutions were measured using a digital viscometer—Brookfield model RV DV-II+, with the Rheocalc V3.1-1 program—at 20, 25, 30, 35 and 40 °C.

A Biolar ZPO polarizing microscope equipped with an advanced image analysis system (MultiScan V. 14.02) was used to prepare images of the aqueous solution of chitosan containing HAp and β-TCP particles.

Images of the cross-section and surface of the fibers were obtained using a scanning electron microscope—SEM/ESEM, Quanta 200 (W), FEI Co., USA.

The size of the nanoparticles of HAp, β-TCP and HAp/β-TCP in chitosan solution was measured using a ZETASIZER 2000 (Malvern Instruments) apparatus.

The amount of chitosan in the spinning solutions was determined using the gravimetric method. A film was formed from a weighed amount of the solution by evaporation of water and drying at 60 °C. This was then coagulated in an aqueous solution of 3.0 wt% sodium hydroxide, rinsed and dried to constant weight at 105 °C.

The water retention value (WRV) was determined according to Standard ISO/FDIS 23714.

The mechanical properties of the chitosan fibers were tested according to Standards PN-ISO-1973:1997 and PN-EN ISO 5079:1999 in an air-conditioned room at 65 ± 4% relative humidity and 20 ± 2 °C.

Spectrophotometric spectra in the infrared range were prepared using FTIR apparatus produced by Unicam Co. equipped with the control program Winfirst ATI Mattson. Samples were prepared in the form of pressed cubes in potassium bromide (KBr Aldrich Co.).

Samples of the fibers were mineralized at 575 °C in 6 M HCl to determine the calcium content, which was measured in the mineralized residue by flame atomic absorption spectrometry (FAAS) at a wavelength of 422.7 nm. A SCAN-1 f-my Thermo Jarrell Ash atomic absorption spectrometer was used for the analysis.

The ash content in the chitosan fibers was estimated according to standard EN-ISO3451-1.

## 4. Conclusions

The presence of HAp/β-TCP nanoparticles in the solution of chitosan had a beneficial effect on the production of the modified chitosan fibers. It allowed a smooth run in the spinning process at a speed of 31 m/min. The addition of the HAp or β-TCP particles resulted in a decrease in the tenacity of the modified chitosan fibers. Only the complex of chitosan with HAp/β-TCP nanoparticles had the same tenacity as regular chitosan fibers. Chitosan fibers were prepared with a calcium content of 14.35 g/kg and an ash content of 4.8%. The HAp/β-TCP-modified chitosan fibers had higher WRV values than the regular ones. Their more hydrophilic character may contribute to the higher susceptibility of fibers to enzymatic degradation.

When we compare the HAp/β-TCP-modified chitosan fibers with the regular chitosan fibers (Chit 58) we found a similar mechanical properties with higher content of calcium, that is the main mineral responsible for hard tissue regeneration. The nanoparticles of ceramic calcium phosphates in the modified chitosan fibers could probably improve the speed of the release of minerals.

This research also shows a method to obtain ceramic nanoparticles in chitosan solution from commercial calcium phosphates.

## Figures and Tables

**Table 1:** Some properties of chitosan solutions containing hydroxyapatite (HAp) and tricalcium phosphate (β-TCP) particles.

Solution code	Percentage of solution used, %			Concentration of				Dynamic viscosity/temp.

				β-TCP	HAp	Acetic acid	Chitosan	

	A	B	C	wt%	wt%	wt%	wt%	Pa/°C
Chit 58	100	-	-	-	-	3.00	5.16	19000/52
MCT 6	83.3	16.7	-	0.333	-	2.75	4.50	7500/49
MCT 7	83.3	-	16.7	-	0.330	2.75	4.63	9250/49
MCT 8	71.4	14.3	14.3	0.286	0.283	2.36	4.46	4500/52
MCT 11	62.5	25.0	12.5	0.707	0.252	2.10	4.01	1750/51

A chitosan solution (A) is 5.0 wt% chitosan was prepared in aqueous 3.0 wt% weight acetic acid; Solution B was an aqueous 2.0 wt% solution of chitosan in 0.9 wt% solution of hydrochloric acid with a 2.0 wt% weight content of β-TCP nanoparticles; Solution C was a diluted aqueous 2.5 wt% chitosan solution in 1.5 wt% acetic acid containing HAp particles at a concentration of 2.0 wt%.

**Table 2:** Selected parameters of chitosan solutions containing HAp, β-TCP and HAp/β-TCP.

Chitosan solution	Range of particle size	Size of fraction with highest volume content	Percentage of volume	Potential Zeta

	nm	nm	%	mV
Solution B	28.9–164.9	65.2	11.5	43.0 ± 2.3
Solution C	417.3–1495	745.4	34.2	45.3 ± 2.3
Blend of chitosan solutions B/C in 2:1 ratio	12.8–58	22.9	19	52.9 ± 4.0

**Table 3:** Impact of HAp, β-TCP and HAp/β-TCP concentration in the chitosan solution on the mechanical properties of fibers.

Parameter		Chit 58	MCT 6	MCT 7	MCT 8	MCT 11
Linear density	dtex	4.39	4.48	5.14	5.41	4.16
Coefficient of variability of linear density	%	1.25	2.48	1.93	3.57	1.64
Confidence interval of linear density	%	±1.55	±3.08	±2.40	±4.43	±2.04
Breaking force	cN	3.61	3.51	2.46	2.91	3.35
Coefficient of variability of breaking force (conditioned)	%	14.6	14.0	32.2	28.6	19.5
Confidence interval of breaking force	%	±6.02	±5.77	±13.3	±11.8	±8.03
Tenacity (cond)	cN/tex	8.22	7.83	4.79	5.38	8.05
Elongation at break (cond)	%	17.0	22.0	9.9	11.0	12.0
Breaking force (wet)	cN	2.80	2.21	1.78	2.48	2.25
Coefficient of variability of breaking force (wet)	%	36.8	31.9	49.9	33.4	18.3
Tenacity (wet)	cN/tex	6.38	4.93	3.46	4.58	6.86
Elongation at break (wet)	%	7.8	7.3	7.8	6.1	8.00

**Table 4:** Water retention value (WRV) content of calcium and ash in HAp/β-TCP modified chitosan fibers.

Fiber code	WRV	Ash	Calcium content

	%	%	g/kg
Chit 58	158	0.1	0.14
MCT 6	163	0.4	0.35
MCT 7	154	3.2	8.45
MCT 8	331	4.8	9.95
MCT 11	210	4.8	14.35

Cross-sections of selected HAp/β-TCP-modified chitosan fibers are illustrated in SEM images in Figure 5.
